# Nucleotide sequence analyses of the *MRP*1 gene in four populations suggest negative selection on its coding region

**DOI:** 10.1186/1471-2164-7-111

**Published:** 2006-05-10

**Authors:** Zihua Wang, Pui-Hoon Sew, Helen Ambrose, Stephen Ryan, Samuel S Chong, Edmund JD Lee, Caroline GL Lee

**Affiliations:** 1 Department of Biochemistry, National University of Singapore, Singapore; 2 Graduate Programme in Bioengineering, National University of Singapore, Singapore; 3 Division of Medical Sciences, National Cancer Center, Singapore; 4 AstraZeneca, Alderley Park, Macclesfield, UK; 5 AstraZeneca, Wilmington, DE, USA; 6 Departments of Pediatrics & Obstetrics/Gynecology, Singapore; 7 Departments of Pediatrics and Gynecology & Obstetrics, and McKusick-Nathans Institute of Genetic Medicine, The Johns Hopkins University School of Medicine, Baltimore, MD, USA; 8 Department of Pharmacology, National University of Singapore, Singapore

## Abstract

**Background:**

The *MRP*1 gene encodes the 190 kDa multidrug resistance-associated protein 1 (MRP1/ABCC1) and effluxes diverse drugs and xenobiotics. Sequence variations within this gene might account for differences in drug response in different individuals. To facilitate association studies of this gene with diseases and/or drug response, exons and flanking introns of *MRP*1 were screened for polymorphisms in 142 DNA samples from four different populations.

**Results:**

Seventy-one polymorphisms, including 60 biallelic single nucleotide polymorphisms (SNPs), ten insertions/deletions (indel) and one short tandem repeat (STR) were identified. Thirty-four of these polymorphisms have not been previously reported. Interestingly, the STR polymorphism at the 5' untranslated region (5'UTR) occurs at high but different frequencies in the different populations. Frequencies of common polymorphisms in our populations were comparable to those of similar populations in HAPMAP or Perlegen. Nucleotide diversity indices indicated that the coding region of *MRP*1 may have undergone negative selection or recent population expansion. SNPs E10/1299 G>T (R433S) and E16/2012 G>T (G671V) which occur at low frequency in only one or two of four populations examined were predicted to be functionally deleterious and hence are likely to be under negative selection.

**Conclusion:**

Through *in silico *approaches, we identified two rare SNPs that are potentially negatively selected. These SNPs may be useful for studies associating this gene with rare events including adverse drug reactions.

## Background

The development of drug resistance poses a serious limitation to the effective treatment of cancer. Although several different drug resistance mechanisms have been described, members of the ABC transporter superfamily have generated great interest because of their contribution to multidrug resistance of tumor[1,2]. The 170 kDa P-glycoprotein, encoded by the *MDR*1 gene, was the first member of this family to be described [3]. Subsequently, the 190-kDa multidrug resistance-associated protein-1 (MRP1/ABCC1) was isolated from a multidrug resistance lung cancer cell line that does not express *MDR*1 [4]. Both these transporters have been implicated in the resistance of various cancers to chemotherapy. Although MRP1 is only 18% identical to MDR1 at the amino acid level, it transports several similar drugs as MDR1 including doxorubicin, vincristine and colchicine. However, while drugs transported by MDR1 are usually neutral or cationic, drugs effluxed by MRP1 are anionic, frequently conjugated with glutathione and other anions, or are co-transported with glutathione [2]. MRP1 has also been implicated to play important roles in cellular anti-oxidative defense and inflammation [5,6].

*MRP*1 is located on chromosome 16 at band 13.1 and spans approximately 200 kb. It contains 31 exons and encodes 1531 amino acids. The MRP1 protein is predicted to comprise three membrane spanning domains (MSDs) and two nucleotide binding domains (NBDs) [4-7].

Genetic polymorphisms in *MDR*1 have been associated with differences in MDR1 expression and function as well as drug response and disease susceptibilities [8-10]. SNPs within *MDR*1 that have been associated with functional differences were found to demonstrate evidence of recent positive selection [11]. However, less is known about the polymorphisms within *MRP*1. Although numerous SNPs have been identified within this gene.([12-17], most of these studies were performed on a single population which was primarily either Chinese, Japanese or Caucasians in origin. Thus far, no association have been observed between the few SNPs at *MRP*1 and functional differences [12,17-19] possibly because neither the functionally important SNP nor SNPs in LD with the functional SNP were examined. These studies which examined only a few of the many SNPs within *MRP*1, without knowledge of the functional SNP nor the LD or haplotype profile in that population may not have been powerful enough to identify any association. Recently, we found evidence of genomic signatures of recent positive selection in a SNP at the 5' flanking region (5'FR) of *MRP*1 in a Caucasian population and demonstrated that this SNP altered *MRP*1 promoter activity [20].

In the present study, we sequenced all the exons as well as the 5' and 3' flanking regions of *MRP*1 to comprehensively scan for polymorphisms in 142 DNA samples from four different populations, namely, the Chinese, Malays, Indians and Caucasians. Nucleotide diversity of the exonic polymorphisms was determined and the functional effects of the non-synonymous SNPs were predicted using three programs, SIFT, PolyPhen and PANTHER. We found that SNPs E10/1299G>T, which resulted in arginine-serine substitution at amino acid position 433 (R433S) and E16/2012G>T, which resulted in glycine-valine substitution at amino acid position 671 (G671V), may potentially adversely affect the function of MRP1. While these two SNPs, which have low minor allele frequencies (<3%), may not be useful for studies associating this gene with common diseases/drug response, it may, nonetheless, be useful for studies associating this gene with rare events including adverse drug reactions (ADRs).

## Results and discussion

### Profile of polymorphisms within *MRP*1 in the different populations

**Figure 1:**
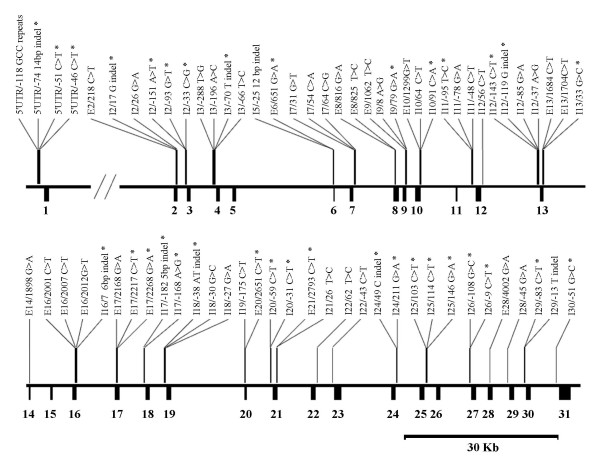
**Distribution of polymorphisms identified in this study across *MRP*1**. The respective positions of all the polymorphisms across *MRP*1 are displayed. Polymorphisms at this locus include 60 SNPs, ten indels and one short tandem repeat (STR). Polymorphisms that have not been previously reported are highlighted with asterisks.

*De novo *sequencing of approximately 18 kb of genomic DNA at *MRP*1, including all the 31 exons as well as flanking regions, was performed in 142 healthy individuals from four different populations to identify polymorphisms at *MRP*1 in the different populations. A total of 71 polymorphisms were identified including 60 bi-allelic SNPs, ten indels and one short tandem repeat (STR) (Figure 1, Tables 2, 3, 4). An examination of currently reported SNPs in the dbSNP Build 125 database [21] and published reports [12-17,22] revealed that 26 SNPs and 8 indels were not previously reported and hence represent novel polymorphisms.

Nineteen of the 60 SNPs identified were found in all four populations while 28 of these SNPs were population-specific with 22 of these population-specific SNPs occurring only once out of 284 chromosomes examined (singletons). While the STR and two indels were polymorphic in all the populations examined, the other seven indels were population-specific of which six were singletons.

None of the indels or STR identified occurred within exons (Fig. 1, Tables 3 and 4). Eighteen of the 60 bi-allelic SNPs were found in exonic regions, six of which resulted in non-synonymous change (Fig. 1, Table 2). These results suggest that polymorphisms at *MRP*1 are largely conservative since less than 10% of these polymorphisms (6/71) presented as non-synonymous changes which are potentially capable of disrupting the MRP1 protein structure/function. Nonetheless, it is possible for synonymous or intronic SNPs to affect MRP1 expression or function through the alteration of the mRNA transcript stability or folding [23] thereby affecting downstream splicing[24,25], processing[26], translational control [27] or regulation [28]. Additionally, polymorphisms at the 5'UTR/promoter and 3'UTR may influence promoter activity and hence gene expression or mRNA transcript stability.

Interestingly, although no polymorphisms were identified at the 3'UTR region (exon 31), four polymorphisms, including the STR (Table 4) and one indel (Table 3) were found to reside at the 5'UTR/reported core promoter region [29] of *MRP*1. Three of these promoter polymorphisms were novel but population-specific with SNPs 5'UTR/-46C>T and 5'UTR/-51C>T occurring only in the Malay population and the polymorphism 5'UTR/-74 14 bp indel occurring only in the Indian population. Insertion/deletion polymorphisms in promoter regions have been correlated with the modulation of the expression of genes (e.g. matrix metalloproteinase I gene [30]). It is thus possible that the 14 bp indel polymorphism in the Indian population may influence the promoter activity and hence the expression of *MRP*1.

The STR polymorphism found at the 5'UTR/promoter region of *MRP*1 is a GCC trinucleotide repeat and 7–16 of such repeats were observed in the four populations (Table 4). The most commonly occurring GCC repeat number in all the four populations was 13 which occurred at a frequency of 50.00%, 51.43%, 47.14% and 56.94% in the Chinese, Malay, Indian and Caucasian populations, respectively. Eleven GCC repeats also occurred at high frequencies (≥ 20%) in most of the populations except the Caucasians. Interestingly, while seven GCC repeats occurred at relatively high frequencies in the Indian and Caucasian populations (≥ 12%), this number of repeats was not observed in either the Chinese or Malay population. These observations highlight the differences in the distribution of the number of the *MRP*1 promoter GCC repeats in the different populations with the Indians and Caucasians being more similar to each other than to the Chinese and Malays. The number of STR repeats residing within or close to promoters has been found to modulate the promoter activity of genes [31-34]. Interestingly, differences in the CGG and GCC trinucleotide repeats at the 5'UTR/promoter region of the Fragile X mental retardation genes (*FMR*1 and *FMR*2, respectively) have been associated with differences in the methylation status of the promoter and expression of the genes [35]. Hence this common polymorphism at the 5'UTR/promoter region of *MRP*1 with distinctly different distribution of repeat numbers in the different population may have potential functional significance.

### Comparison of polymorphisms identified in this study with those reported in the HapMap and Perlegen databases

Two publicly available databases HapMap [36] and Perlegen [37] examined genome-wide polymorphisms (including polymorphisms at the *MRP*1 gene) in several populations. HapMap genotyped already known SNPs from public databases at a density of approximately one SNP per 5 kb of DNA in 4 different populations namely, 45 Japanese from Tokyo, 45 Chinese from Beijing (CHB), 60 US residents with northern and western European ancestry by the Centre d'Etude du Polymorphisme Humain (CEPH) and 60 Yoruba people of Ibadan (YRI). Approximately 1.6 million SNPs from 24 Han Chinese (CH), 24 European American (EA) and 23 African American (AA) were successfully genotyped in the Perlegen project. The SNPs genotyped in the Perlegen project were either reported in public databases or identified through their array-based re-sequencing of 24 human samples of diverse ancestry [38,39]. We thus compared the polymorphisms at the MRP1 gene that we identified through *de novo *sequencing of DNA samples from 142 individuals of 4 different populations with those reported in the HAPMAP and Perlegen databases. Of the populations examined in HapMap and Perlegen, only two populations, namely the CHB and CEPH/EA, were similar to the populations that we studied. As shown in Table 5A, only 19 and 14 polymorphisms that we identified were also genotyped in the HapMap and Perlegen projects. Curiously, 25/23 and 1/1 polymorphisms reported in HapMap and Perlegen, respectively, were found to be monomorphic in similar populations that we examined. Nonetheless, all the SNPs examined in the two databases that did not occur in our populations were found to be either monomorphic or of low frequency (<5%) in similar populations examined in the two databases (Table 5A). On the other hand, 41/41 and 46/46 polymorphisms that we identified were not examined in either the HapMap or the Perlegen project, respectively. While many of these polymorphisms were of low frequencies or were monomorphic in the two populations that were similar to the HapMap/Perlegen populations, 8 of these polymorphisms were found to be of relatively high frequencies (>5%) in at least one of the two populations. Some of the low frequency polymorphisms represent novel SNPs identified in this study.

Polymorphisms in our study that was also genotyped in the HapMap and Perlegen projects were found to have similar frequencies in similar populations (Table 5B). Paired T-test revealed no significant difference (P > 0.05) between allele frequencies in the respective populations from our study and those from the HapMap or Perlegen database (Table 5C). Interestingly, significant difference (P < 0.05) was observed between data obtained from the HapMap database and those from the Perlegen database especially for the Chinese population probably due to fewer samples being examined in the Perlegen database.

### Nucleotide diversity at *MRP*1

The extent of variation at *MRP*1 was evaluated using two conventional measures of nucleotide diversity: π, the average heterozygosity per site and θ, the population mutation parameter[40]. Tajima's D statistic was also calculated to assess deviation from the neutral mutation model[41]. A positive Tajima's D value for a single gene is indicative of positive heterozygote advantage while a negative Tajima's D value for an individual gene suggests selection of a specific allele over the alternative allele(s)[42]. However, when a negative Tajima's D value is observed in most of the genes that were examined in a particular population, it is suggestive of a recent expansion in that population[42].

With all the exonic regions sequenced, the above nucleotide diversity statistics were determined for non-synonymous versus synonymous SNPs at *MRP*1 (Table 6). The θ value for synonymous SNPs at *MRP*1 was found to be 16.15 × 10^4 ^(Table 6) which was comparable to mean θ values of other reported genes including 24 transporter genes (20.14 ± 4.10) × 10^4 ^[43], 75 candidate genes associated with blood pressure homeostasis (15.1 ± 3.6) × 10^4 ^[44] but slightly higher than the mean θ values of 106 random genes (10.03 ± 2.52) × 10^4 ^[45]. However, the θ value for non-synonymous SNPs (11.73 × 10^4^) at *MRP*1 was much higher than mean θ values of the other reports (3.59 ± 0.90 to 5.7 ± 1.4) × 10^4 ^[43-45] probably due to the small size of the *MRP*1 exons in which the non-synonymous SNPs reside. Interestingly, while the π of synonymous SNPs (π_s_) at *MRP*1(12.62) was comparable to the reported mean π_s _values in other genes (9.73 ± 4.86 to 10.67 ± 5.07) × 10^4 ^[43,45], the π_ns _at *MRP*1 (0.94) was much lower than the mean reported π_ns _values for the other genes (2.20 ± 1.12 to 2.75 ± 1.31) × 10^4 ^[43,45]. This low π_ns _at *MRP*1 was also reported previously [43] with the reported π_ns _value (0.15) being much lower than the present observation (0.94). Notably, the π_ns_/π_s _at *MRP*1 was less than 1 (0.0743 in this study and 0.0110 in the previous study [43]), suggesting that this gene is likely to be under selective pressure. Importantly, the θ values for both synonymous and non-synonymous SNPs were greater than the corresponding π values, resulting in negative Tajima's D statistic which suggests that the coding region of *MRP*1 may have undergone negative selection or population expansion. It is more likely that *MRP*1 gene have undergone negative selection since the average total nucleotide diversity in the *MRP*1 gene (π_total_) (9.25) was found to be greater than the amino acid diversity (π_ns_) (0.94) [43].

### SNPs E10/1299G>T and E16/2012 G>T are potentially deleterious

As nucleotide diversity statistics suggest that the coding region of *MRP*1 may be under negative selection, we thus further analyzed the exonic SNPs at *MRP*1 to evaluate if any of these SNPs may have deleterious effects on MRP1 structure/function.

**Figure 2:**
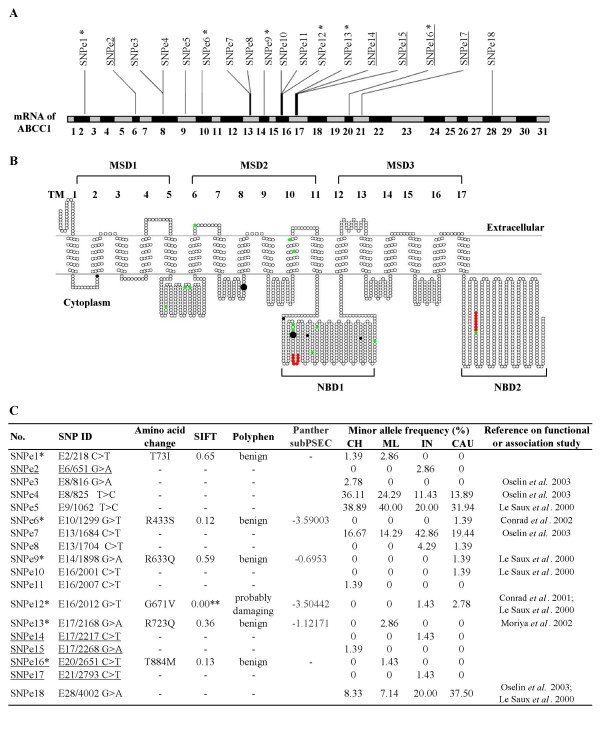
**Profiles of the 18 exonic SNPs in *MRP*1**. (A) The distribution of all the exonic SNPs on the mRNA of *MRP*1 is presented. (B) The topological model of MRP1 protein secondary structure is predicted using the SOSUI program and the positions of the SNPs on the topological image are displayed using the TOPO2 program. Approximate locations of predicted individual transmembrane helices, membrane spanning domains and nucleotide binding domains are indicated as TM 1-17, MSD 1-3 and NBD1-2, respectively. The six nonsynonymous SNPs are highlighted in black while the 12 synonymous SNPs are colored green. Consensus sequences for Walker A and B are highlighted in red. The two nonsynonymous SNPs predicted by PANTHER to be potentially deleterious are highlighted by large black dots. (C) Table showing *in silico *prediction of functional significance of exonic SNPs and their frequencies in the different populations. SNPs that have previously been utilized for association/functional studies are also presented. For PANTHER prediction, if the subPSEC score is lower than -3.5, it can be interpreted that the amino acid change could have high probability of deleterious functional effect. Note: Underlined SNPs represent SNPs that have not been previously reported. * The six nonsynonymous SNPs are highlighted with single asterisks. ** This SNP is predicted to have effect on the function of the protein.

Exonic SNPs, particularly non-synonymous SNPs, have the potential to alter the secondary/tertiary structure of proteins and/or affect the protein function. A total of 18 exonic SNPs were identified at this gene locus of which five have not been previously reported (Fig. 2A, C). Most of the exonic SNPs occurred at low frequencies (<5%) in only one or two populations. While at least 30% of the synonymous SNPs at *MRP*1 occurred at greater than 5% frequency in all the four populations examined, all of the non-synonymous SNPs occurred at less than 3% in only one or at most two populations (Fig. 2C). This observation highlights the conservation of exonic polymorphisms at *MRP*1 and suggests that altering the non-synonymous SNPs may have a deleterious effect and are likely to be selected against, resulting in their low frequencies.

To assess if any of the non-synonymous SNPs at *MRP*1 have potentially damaging effect on the protein structure/function, the location of these six SNPs were displayed on the MRP1 protein topological image using the SOSUI and TOPO2 programs. As evident in figure 2B, none of the non-synonymous SNPs reside in the transmembrane regions although four of these SNPs reside near or within the nucleotide binding domain (NBD) of the MRP1 protein. Nonetheless, SNPe1 (SNP e2/218 C>T) and SNPe6 (SNP e10/1299G>T) reside near the transmembrane region, while SNPe12 (SNP E16/2012 G>T) reside on a conserved glycine residue near the conserved Walker A consensus motif of the NBD [12], suggesting that these SNPs may have functional significance. SNP e2/218 C>T was only found at less than 3% in the Chinese and Malay populations while SNP E10/1299 G>T occurred at less than 2% in the Caucasian population only and SNP E16/2012 G>T occurred at less than 3% in the Indian and Caucasian populations (Fig. 2C). The SNP frequencies of SNP E10/1299 G>T and SNP E16/2012 G>T in the Caucasian population were comparable to a previous report [12].

Three different algorithms, SIFT[46], Polymorphism Phenotyping (PolyPhen) [47]and PANTHER [48]were then utilized to predict the functional significance of the six non-synonymous SNPs. SIFT predicts the effect of amino acid substitutions based on the assumption that the important amino acid will be conserved in the protein family[46]. PolyPhen predicts the effect of the amino acid variant on the function or structure of the protein based on current knowledge of protein structure, interactions and evolution[47] while the PANTHER program predicts the effect of an amino acid substitution on the protein's function using amino acid substitution scores derived from an alignment of related protein sequences and statistics from hidden Markov models[48].

Interestingly, SNP E10/1299 G>T, which is located near the transmembrane domain, was predicted to be potentially deleterious by the PANTHER but not the SIFT or PolyPhen algorithms. This SNP was reported to affect the ability of MRP1 to confer drug resistance as well as to transport organic anions [49] suggesting that the PANTHER program may be more accurate in predicting the functional impact of polymorphisms than SIFT or PolyPhen. This observation is similar to a previous report that utilizes both bioinformatics and biochemical approaches to compare the accuracy of the PolyPhen and PANTHER programs in predicting functionally deleterious polymorphisms in the *ABCA*1 gene [50]. They found that the PANTHER software is significantly (P < 0.05) more accurate in its prediction of the functional consequence of nonsynonymous SNPs. They also reported that the PANTHER program is capable of correctly predicting the functional impact of greater than 94% of the polymorphisms examined while PolyPhen is only ~88% accurate in predicting the functional impact of polymorphisms [50].

Significantly, all of the three different algorithms predicted that SNP E16/2012 G>T, which resides close to Walker A and results in G671V substitution, was likely to have a potentially deleterious effect on protein function (Fig. 2C). The significance of this polymorphism has also been demonstrated previously by Conrad *et al*. [12] who reported that the mRNA expression of peripheral lymphocytes from individuals carrying the SNP E16/2012 G>T polymorphism was lower than the average expression level. The lower expression of the MRP1 G671V transcript is suggestive of greater accumulation of MRP1 drugs in the cells which may lead to adverse drug reactions. Curiously, that report also found that the G671V polymorphism did not affect the transport of MRP1 substrates including leukotriene C_4_, 17β-estradiol 17β-(D)-glucuronide and estrone sulfate by membrane vesicles prepared from transiently transfected HEKSV293T cells [12]. Recently, the same group also reported similar MRP1 protein expression levels and transport properties in human embryonic kidney cells were transfected with MRP1 constructs carrying either glycine or valine at amino acid position 671 [51]. The observation that the G671V polymorphism did not affect MRP1 protein expression or transport ability of some MRP1 substrates *in vitro *[12,51] does not rule out the possibility of functional significance of this polymorphism *in vivo *especially since the same group reported decreased transcript expression in individuals carrying this polymorphism. It is still possible that this polymorphism affect the transport of other MRP1 substrates that has not been examined. It is also possible that although the SNP E16/2012 G>T polymorphism does not affect MRP1 transport ability, it may affect other yet-to-be-examined functional properties of the protein (e.g. drug resistance capability or cellular anti-oxidative defense or inflammation). It has been reported that an artificial mutation E1089Q created in *MRP*1 markedly affected the ability of MRP1 protein to confer resistance without affecting its ability to transport organic anions [52]. Hence, the SNP E16/2012 G>T polymorphism warrants further investigation.

Hence, the bioinformatics approach may be useful in facilitating the prediction of potentially functionally significant polymorphism so that future research may be directed to characterizing these polymorphisms.

### Functional implications of polymorphisms at *MRP*1

The current detailed characterization of polymorphisms at *MRP*1 in four different ethnic populations highlights several characteristics about this gene that may facilitate more rational approaches to studies associating this gene with functional changes. We have previously reported that the diverse haplotypes and weak LD across *MRP*1 [20]could perhaps provide an explanation for the failure of previous studies to detect association between polymorphisms in this gene and functional differences [12,17-19] and highlight the importance of fully characterizing the LD and haplotype profiles of the gene before embarking on association studies. Its LD and haplotype architecture suggest that it may be necessary to identify alternative approaches for association studies of this gene as it may not be feasible to utilize tag SNPs. A possible approach is to identify polymorphisms with potential functional significance before performing association studies, possibly by identifying those polymorphisms that may have been subjected to selection pressures.

We recently identified a high frequency SNP at the 5' flanking promoter region of *MRP*1 that demonstrated evidence of recent positive selection and affected the promoter activity of *MRP*1[20]. In this report, through the sequencing of the *MRP*1 exonic and flanking regions, we identified a GCC-trinucleotide multi-allelic STR polymorphism residing within the 5'UTR/promoter region of *MRP*1 that was found at relatively high frequencies in all populations examined. Notably, the frequency distribution of the different number of STR alleles in the different population was found to be different (Table 4). Although it was previously reported that the 5'UTR/promoter region contains the GCC-triplet repeats that is absent in the rodent sequence and 7, 13 and/or 14 of these repeats were observed in different cell lines and PBMC from a single individual [7,53], no reports have yet examined the variation of this polymorphism in the different ethnic populations. This STR is approximately 296 bp from the SNP that we previously reported to show evidence of recent positive selection[20]. Given that the selection is recent it may be expected that it would be in strong LD with the positively selected SNP. Since STRs within/near promoters have been implicated to affect promoter activity and expression levels of the gene, it would be worthwhile to further examine the effect that this polymorphism together with the positively selected SNP have in influencing promoter activity and hence expression of *MRP*1.

Interestingly, while the promoter region of *MRP*1 may be under recent positive selective pressure[20], in this study we also found that the coding region of this gene may have undergone negative selection pressure as suggested by nucleotide diversity indices. Two coding SNPs E16/2012 G>T and E10/1299 G>T have been predicted by either the PANTHER program or all three programs (SIFT, PolyPhen and PANTHER) to have a deleterious effect on the structure/function of the protein. The significance of these SNPs for general association studies may be limited since these SNP occurs at very low frequencies (<3%) in only one or two of the four populations examined. Nonetheless, this SNP may be associated with rare events including ADR.

## Conclusion

In summary, based on the "common disease-common variant" hypothesis, the previously reported common polymorphism within the promoter of *MRP*1 that showed evidence of recent positive selection [20]would be useful for association studies of common diseases/drug response. Nonetheless, the rare exonic SNP(s) in this gene that we demonstrate here to be likely to be under negative selection pressure may be useful for studies associating this gene with rare phenotypes including ADR, which has been listed as the top five leading causes of death in Western countries [54].

## Methods

### Study population

The populations examined include individuals residing in Singapore from the following ethnic groups: 36 Caucasians and Chinese as well as 35 Malays and Indians. Race and ethnic group were declared by the volunteers to be true to three generations. Informed consent from the volunteers and ethical approval from the National University Hospital and the Changi General Hospital Institutional Review Boards were obtained.

### PCR and DNA sequencing

The *MRP*1 genomic DNA (NT_0101393.13) sequence was obtained from GenBank [55] and used as the reference sequence. For the sequencing of all 31 exons of *MRP*1, 30 pairs of primers (see Table 1) were designed using Vector NTI 7.0 software and utilized to amplify these exons. The amplicons spanned the entire exon as well as some flanking sequences, to ensure that the splice donor and acceptor sites were also included. The PCR reaction was performed in a 10 μl volume reaction containing 40 ng genomic DNA template from the above mentioned samples, 5 μl 2 × PCR master mix buffer (Qiagen, Valencia, CA, USA), with or without 1 μl Q-solution (depending on the GC content of the amplicon) as well as 0.20 μM/L of sense and anti-sense primers. PCR was carried out in a GeneAmp^® ^PCR System 9700 (Applied Biosystems, Foster City, CA) with the thermal cycling conditions as follows: an initial denaturation at 94°C for 15 min followed by 35 cycles at 94°C for 30 sec, temperature for the optimal annealing of each amplicon as specified (Table 1) for 90 sec, and extension at 72°C for 60 sec. This was then followed by a final elongation step at 72°C for 10 min. The PCR products obtained were then treated with exonuclease I and shrimp alkaline phosphatase (SAP, United States Biochemical). Sequencing reactions were performed using ABI PRISM Big Dye Terminator (V3.0) kit and the conditions for the sequencing reactions were (for all the exons except exon 1): 94°C for 15 min followed by 30 cycles at 96°C for 10 sec, 50°C for 5 sec and 60°C for 4 min. Due to the high GC content of exon 1, the sequencing conditions were modified as follows: 94°C for 15 min followed by 35 cycles at 98°C for 30 sec, 48°C for 10 sec and 60°C for 5 min. The final product was resolved by automated capillary electrophoresis on an ABI PRISM 3700^® ^DNA analyzer (Applied Biosystems). The DNA sequence of each exon obtained experimentally was then aligned against the reference sequence (NT_0101393.13) using the Vector NTI 7.0 software to identify the polymorphic sites. Polymorphisms identified were verified through bi-directional re-sequencing of all samples whose chromatograms do not clearly display the polymorphism as well as randomly selected samples whose chromatograms clearly show the polymorphism.

### Population genetic parameters

Two common parameters of nucleotide diversity were calculated: the neutral parameter (θ) which is the estimate of population mutation parameter based on the number of polymorphic sites in the samples [40] and nucleotide diversity (π) which is the direct estimate of heterozygosity per site, or the average proportion of nucleotides that differ between any randomly sampled pair of sequences [40]. Each of these two parameters was calculated for synonymous and nonsynonymous SNP sites. Tajima's D statistic was also calculated to assess deviations from the neutral mutation model [41].

### *In Silico *characterization of polymorphisms in exons

The programs Sorting Intolerant From Tolerant (SIFT) [46], Polymorphism Phenotyping (PolyPhen) [47] and PANTHER [48] were utilized to evaluate the potential effect of amino acid substitutions resulting from the polymorphisms. Since the position of the non-synonymous polymorphic amino acid residue on the MRP1 protein may provide important clues with regards to its potential functionality, the SOSUI program [56] was utilized to predict the topology of the MRP1 protein and the TOPO2 program [57] was used to display the location of the SNPs on the MRP1 protein topological image.

## Authors' contributions

ZW contributed to the design of the experiments, data analysis and the write-up of the manuscript. PHS carried out the sequencing experiments. HA and SR contributed to the critical review of the draft manuscript. SSC and EJDL contributed to the conception and critical review of the draft manuscript. CGL (corresponding author) conceived the study, and contributed to the design of experiments, coordination, critical evaluation of the data and analyses as well as the final writing of the manuscript. All the authors have given the final approval of the version to be published

## Figures and Tables

**Table 1:** Primers and PCR conditions for amplifying and sequencing *MRP*1

**Exons**	**Primer Direction**	**Length (bp)**	**Primer Sequence (5' – 3')**	**Amplicon Length(bp)**	**Additional Primer For Sequencing**	**Tm**
Exon 1	Forward	22	GCATTTGAAAAGTGGTCGCAGG	688		59
	Reverse	20	TCCGCAGGAACTGAGTCACC			
Exon 2	Forward	20	GCAGAAGACACCACATACCT	510		60
	Reverse	20	AGAAGAAGGAACTTAGGGTC			
Exon 3	Forward	19	GCATGGTGACCAGACAAAC	501		60
	Reverse	19	CTCCAGCTGATCATTGCCT			
Exon 4	Forward	21	ACGTGGTCCATTAAGAAATAG	571		61
	Reverse	20	GACTTCTACACAAGCCAGAG			
Exon 5	Forward	19	CCCAGCCCCAGAATGTGAT	358		58
	Reverse	18	CCCCAGCCACATCTAAGC			
Exon 6	Forward	22	TGTTGTATTGTGGTTGCACATG	383		60
	Reverse	20	GAGCTGAGCATGTTCATTCG			
Exon 7	Forward	20	TCCCTAAGTCTTTTGTATGC	574	AAGCCATTTTTCCTGCATGAC (forward)	61
	Reverse	19	TACCCCATTTGGCAGAAAA			
Exon 8	Forward	20	AGAGAGCTTAAGGACCTTGT	591		59
	Reverse	19	TATGAGCCCACTTCAGGAC			
Exon 9	Forward	19	CGTGTTCCCTATGCAATTC	602		59
	Reverse	18	CCTGCCACCTAAGGTCAC			
Exon 10	Forward	18	TCCTGGGCAGACAGATAG	439		61
	Reverse	18	TGAACCACAGCCGGAACT			
Exon 11	Forward	19	GCTTGGGAGAAAGGAGCGT	452		61
	Reverse	19	TGAGTCCAACTGGCAGGCA			
Exon 12	Forward	20	TAATAGACGGTGAAGTTGAG	743	ATGAAGAGCAAAGACAATCG (forward)	61
	Reverse	20	AAGTAATTCTCTTGCCTCAG			
Exon 13	Forward	21	GTCGTTGATTTATCCAGTTCA	523		61
	Reverse	20	CTTTCTTTCAGGCATGACCA			
Exon 14	Forward	20	TCTGAAATACCTTTTGTGGG	627		60
	Reverse	19	GGTCAAAGCCTTGGAAAGT			
Exon 15	Forward	21	TTACAAGGACAAAGCTGCTTG	475		60
	Reverse	19	TGTATCTGCACCCATTGTC			
Exon 16	Forward	20	GTTTAGTACAGTCTTGCCTT	463		60
	Reverse	19	CCAAAATCCTGCCTTCTAG			
Exon 17	Forward	21	GTGGGCCAGCTGTTGTCTCGT	441		61
	Reverse	20	AGTGAGACCTGAGCCACACC			
Exon 18	Forward	18	CGTATTGTGAGTCTCAAG	596	TACCCATTACCACAACTG (reverse)	61
	Reverse	18	TTTTCCGACCCCTCTACC			
Exon 19	Forward	18	GAGTTTTGCCCACCAGGT	462		61
	Reverse	19	GGTGGTTTTCCACATTGCT			
Exon 20	Forward	18	TATGCCCTTTCCCTCATG	758		61
	Reverse	18	GAGCAAAGACCCACACCA			
Exon 21	Forward	19	CTGGAGAGTGACATGGTGG	504		61
	Reverse	20	GGGTGACCCTGAGTAAGTCA			
Exon 22	Forward	20	GTTGAATAGCTAAAGGGGAG	599		61
	Reverse	19	TGTAAAATGGGCACACTGG			
Exon 23	Forward	20	ATGCCTGGTTCATCATTATT	514		58
	Reverse	20	CTTTAGGTAACACTGGTATA			
Exon 24	Forward	20	AGCGTGAGCTATTATTGTCA	727		61
	Reverse	19	ACTCAATAGCAGAGTCGGT			
Exon 25, 26	Forward	19	CCCATTGTGCATGTTTTGA	1442	AGATGCAGCTAAAGCAGTTC* GTGACTGATGGGGTTTCG**	61
	Reverse	20	AAAGACTGGACAAGCGTTAA			
Exon 27	Forward	22	ACCTACTATGTGCTCTGAGCCC	693		61
	Reverse	20	TACTGCCACAGTCCACTCCC			
Exon 28	Forward	18	CGAGTCATTCCTTTTGGG	493		61
	Reverse	20	GTAGGGCCAAATGTATGTTT			
Exon 29	Forward	18	AAGGAGCTCTGATACCCC	405		61
	Reverse	19	GGACAAATGGTCATCTGGG			
Exon 30	Forward	19	CTCCCAAAGTGCTCAGATT	706		61
	Reverse	19	GAAATGCTTGAACCCCAGA			
Exon 31	Forward	21	TCTGAATGTAATGGAACAGTG	1677		61
	Reverse	21	GGAAGAGCATCAGTAACTAAA			

*reverse primer for Exon 25, **forward primer for Exon 26

**Table 2:** Frequencies of single nucleotide polymorphisms (SNPs) identified at *MRP*1

No.*	SNP ID**	**Region**	**Protein residue**	**dbSNP (rs)**	Population***	**n**	**Allele frequency (%)**		**No.**	**SNP ID**	**Region**	**Protein residue**	**dbSNP (rs)**	**Population**	**n**	**Allele frequency (%)**	
							C	T								C	T
**SNPu1**	**5'UTR/-51 C>T**	5'UTR	-	-	CH	36	100.00	0.00	**SNPe4**	**E8/825 T>C**	Exon 8	Val>Val	rs246221	CH	36	36.11	63.89
					ML	35	98.57	1.43						ML	35	24.29	75.71
					IN	35	100.00	0.00						IN	35	11.43	88.57
					CAU	36	100.00	0.00						CAU	36	13.89	86.11
							C	T								C	T
**SNPu2**	**5'UTR/-46 C>T**	5'UTR	-	-	CH	36	100.00	0.00	**SNPe5**	**E9/1062 T>C**	Exon 9	Asn>Asn	rs35587	CH	36	38.89	61.11
					ML	35	98.57	1.43						ML	35	40.00	60.00
					IN	35	100.00	0.00						IN	35	20.00	80.00
					CAU	36	100.00	0.00						CAU	36	31.94	68.06
							C	T								A	G
**SNPe1**	**E2/218 C>T**	Exon 2	Thr>Ile	-	CH	36	98.61	1.39	**SNPi11**	**I9/8 A>G**	Intron 9	-	rs35588	CH	36	61.11	38.89
					ML	35	97.14	2.86						ML	35	60.00	40.00
					IN	35	100.00	0.00						IN	35	80.00	20.00
					CAU	36	100.00	0.00						CAU	36	68.06	31.94
							A	G								A	G
**SNPi1**	**I2/26 G>A**	Intron 2	-	rs8187843	CH	36	0.00	100.00	**SNPi12**	**I9/79 G>A**	Intron 9	-	-	CH	36	0.00	100.00
					ML	35	0.00	100.00						ML	35	0.00	100.00
					IN	35	1.43	98.57						IN	35	1.43	98.57
					CAU	35	7.14	92.86						CAU	36	0.00	100.00
							A	T								G	T
**SNPi2**	**I2/-151 A>T**	Intron 2	-	-	CH	36	98.61	1.39	**SNPe6**	**E10/1299 G>T**	Exon 10	Arg>Ser	-	CH	36	100.00	0.00
					ML	35	100.00	0.00						ML	35	100.00	0.00
					IN	35	100.00	0.00						IN	35	100.00	0.00
					CAU	36	100.00	0.00						CAU	36	98.61	1.39
							G	T								C	T
**SNPi3**	**I2/-93 G>T**	Intron 2	-	-	CH	36	100.00	0.00	**SNPi13**	**I10/64 C>T**	Intron 10	-	rs28363993	CH	36	100.00	0.00
					ML	35	100.00	0.00						ML	35	100.00	0.00
					IN	35	98.57	1.43						IN	35	97.14	2.86
					CAU	36	100.00	0.00						CAU	36	100.00	0.00
							C	G								A	C
**SNPi4**	**I2/-33 C>G**	Intron 2	-	-	CH	36	100.00	0.00	**SNPi14**	**I10/91 C>A**	Intron 10	-	-	CH	36	0.00	100.00
					ML	35	98.57	1.43						ML	35	1.43	98.57
					IN	35	100.00	0.00						IN	35	4.29	95.71
					CAU	36	100.00	0.00						CAU	36	0.00	100.00
							G	T								C	T
**SNPi5**	**I3/-288 T>G**	Intron 3	-	rs4148335	CH	36	2.78	97.22	**SNPi15**	**I11/-95 T>C**	Intron 11	-	-	CH	36	1.39	98.61
					ML	34	1.47	98.53						ML	35	0.00	100.00
					IN	35	15.71	84.29						IN	35	0.00	100.00
					CAU	34	14.71	85.29						CAU	36	0.00	100.00
							A	C								A	G
**SNPi6**	**I3/-196 A>C**	Intron 3	-	rs4148336	CH	36	97.22	2.78	**SNPi16**	**I11/-78 G>A**	Intron 11	-	rs35595	CH	36	20.83	79.17
					ML	35	98.57	1.43						ML	35	27.14	72.86
					IN	35	84.29	15.71						IN	35	11.43	88.57
					CAU	36	84.72	15.28						CAU	36	13.89	86.11
							C	T								C	T
**SNPi7**	**I3/-66 T>C**	Intron 3	-	rs4148337	CH	36	40.28	59.72	**SNPi17**	**I11/-48 C>T**	Intron 11	-	rs3765129	CH	36	88.89	11.11
					ML	35	44.29	55.71						ML	35	91.43	8.57
					IN	35	48.57	51.43						IN	35	97.14	2.86
					CAU	35	74.29	25.71						CAU	36	90.28	9.72
							A	G								C	T
**SNPe2**	**E6/651 G>A**	Exon 6	Ser>Ser	-	CH	36	0.00	100.00	**SNPi18**	**I12/56 C>T**	Intron 12	-	rs17265551	CH	35	98.57	1.43
					ML	35	0.00	100.00						ML	35	98.57	1.43
					IN	35	2.86	97.14						IN	35	94.29	5.71
					CAU	36	0.00	100.00						CAU	36	97.22	2.78
							G	T								C	T
**SNPi8**	**I7/31 G>T**	Intron 7	-	rs8187850	CH	35	100.00	0.00	**SNPi19**	**I12/-143 C>T**	Intron 12	-	-	CH	31	100.00	0.00
					ML	34	100.00	0.00						ML	35	100.00	0.00
					IN	35	98.57	1.43						IN	35	98.57	1.43
					CAU	36	98.61	1.39						CAU	36	98.61	1.39
							A	C								A	G
**SNPi9**	**I7/54 C>A**	Intron 7	-	rs903880	CH	34	1.47	98.53	**SNPi20**	**I12/-85 G>A**	Intron 12	-	rs4148348	CH	36	8.33	91.67
					ML	34	2.94	97.06						ML	35	4.29	95.71
					IN	35	20.00	80.00						IN	35	5.71	94.29
					CAU	36	13.89	86.11						CAU	36	8.33	91.67
							C	G								A	G
**SNPi10**	**I7/64 C>G**	Intron 7	-	rs246232	CH	34	61.76	38.24	**SNPi21**	**I12/-37 A>G**	Intron 12	-	rs35604	CH	36	83.33	16.67
					ML	34	72.06	27.94						ML	35	85.71	14.29
					IN	35	65.71	34.29						IN	35	57.14	42.86
					CAU	35	80.00	20.00						CAU	36	79.17	20.83
							A	G								C	T
**SNPe3**	**E8/816 G>A**	Exon 8	Pro>Pro	rs2230669	CH	36	2.78	97.22	**SNPe7**	**E13/1684 C>T**	Exon 13	Leu>Leu	rs35605	CH	36	83.33	16.67
					ML	35	0.00	100.00						ML	35	85.71	14.29
					IN	35	0.00	100.00						IN	35	57.14	42.86
					CAU	36	0.00	100.00						CAU	36	80.56	19.44
							C	T								C	T

**No.**	**SNP ID**	**Region**	**Protein residue**	**dbSNP (rs)**	**Population**	**n**	**Allele frequency (%)**		**No.**	**SNP ID**	**Region**	**Protein residue**	**dbSNP (rs)**	**Population**	**n**	**Allele frequency (%)**	

**SNPe8**	**E13/1704 C>T**	Exon 13	Tyr>Tyr	rs8187858	CH	36	100.00	0.00	**SNPi28**	**I20/-31 C>T**	Intron 20	-	-	CH	36	100.00	0.00
					ML	35	100.00	0.00						ML	35	100.00	0.00
					IN	35	95.71	4.29						IN	35	100.00	0.00
					CAU	36	98.61	1.39						CAU	36	98.61	1.39
							C	G								C	T
**SNPi22**	**I13/33 I13/33**	Intron 13	-	-	CH	36	0.00	100.00	**SNPe17**	**E21/2793 C>T**	Exon 21	Thr>Thr	-	CH	36	100.00	0.00
					ML	35	0.00	100.00						ML	35	100.00	0.00
					IN	35	1.43	98.57						IN	35	98.57	1.43
					CAU	36	0.00	100.00						CAU	36	100.00	0.00
							A	G								C	T
**SNPe9**	**E14/1898 G>A**	Exon 14	Arg>Gln		CH	36	0.00	100.00	**SNPi29**	**I21/26 T>C**	Intron 21	-	rs11075296	CH	36	0.00	100.00
					ML	35	0.00	100.00						ML	35	0.00	100.00
					IN	35	0.00	100.00						IN	35	1.43	98.57
					CAU	36	1.39	98.61						CAU	36	0.00	100.00
							C	T								C	T
**SNPe10**	**E16/2001 C>T**	Exon 16	Ser>Ser	rs8187863	CH	36	100.00	0.00	**SNPi30**	**I22/62 T>C**	Intron 22	-	rs3887893	CH	36	58.33	41.67
					ML	35	100.00	0.00						ML	35	52.86	47.14
					IN	35	100.00	0.00						IN	35	47.14	52.86
					CAU	36	98.61	1.39						CAU	36	43.06	56.94
							C	T								C	T
**SNPe11**	**E16/2007 C>T**	Exon 16	Pro>Pro	rs2301666	CH	36	98.61	1.39	**SNPi31**	**I22/-43 C>T**	Intron 22	-	-	CH	35	91.43	8.57
					ML	35	100.00	0.00						ML	35	81.43	18.57
					IN	35	100.00	0.00						IN	35	100.00	0.00
					CAU	36	100.00	0.00						CAU	36	100.00	0.00
							G	T								A	G
**SNPe12**	**E16/2012 G>T**	Exon 16	Gly>Val	-	CH	36	100.00	0.00	**SNPi32**	**I24/211 G>A**	Intron 24	-	-	CH	36	6.94	93.06
					ML	35	100.00	0.00						ML	35	4.29	95.71
					IN	35	98.57	1.43						IN	35	1.43	98.57
					CAU	36	97.22	2.78						CAU	36	0.00	100.00
							A	G								C	T
**SNPe13**	**E17/2168 G>A**	Exon 17	Arg>Gln	rs4148356	CH	36	0.00	100.00	**SNPi33**	**I25/103 C>T**	Intron 25	-	-	CH	36	100.00	0.00
					ML	35	2.86	97.14						ML	35	100.00	0.00
					IN	35	0.00	100.00						IN	35	98.57	1.43
					CAU	36	0.00	100.00						CAU	34	100.00	0.00
							C	T								C	T
**SNPe14**	**E17/2217 C>T**	Exon 17	Ser>Ser	-	CH	36	100.00	0.00	**SNPi34**	**I25/114 C>T**	Intron 25	-	-	CH	36	98.61	1.39
					ML	35	100.00	0.00						ML	35	100.00	0.00
					IN	35	98.57	1.43						IN	35	100.00	0.00
					CAU	36	100.00	0.00						CAU	34	100.00	0.00
							A	G								A	G
**SNPe15**	**E17/2268 G>A**	Exon 17	Gly>Gly	-	CH	36	1.39	98.61	**SNPi35**	**I25/146 G>A**	Intron 25	-	-	CH	36	0.00	100.00
					ML	35	0.00	100.00						ML	35	1.43	98.57
					IN	35	0.00	100.00						IN	35	0.00	100.00
					CAU	36	0.00	100.00						CAU	34	0.00	100.00
							A	G								C	G
**SNPi23**	**I17/-168 A>G**	Intron 17	-	-	CH	31	100.00	0.00	**SNPi36**	**I26/-108 G>C**	Intron 26	-	-	CH	36	0.00	100.00
					ML	35	97.14	2.86						ML	35	0.00	100.00
					IN	29	100.00	0.00						IN	35	2.86	97.14
					CAU	32	98.44	1.56						CAU	35	2.86	97.14
							C	G								C	T
**SNPi24**	**I18/-30 G>C**	Intron 18	-	rs2074087	CH	36	15.28	84.72	**SNPi37**	**I26/-9 C>T**	Intron 26	-	-	CH	36	98.61	1.39
					ML	35	12.86	87.14						ML	35	100.00	0.00
					IN	35	37.14	62.86						IN	35	100.00	0.00
					CAU	36	18.06	81.94						CAU	35	100.00	0.00
							A	G								A	G
**SNPi25**	**I18/-27 G>A**	Intron 18	-	rs28363997	CH	36	0.00	100.00	**SNPe18**	**E28/4002 G>A**	Exon 28	Ser>Ser	rs2239330	CH	36	8.33	91.67
					ML	35	0.00	100.00						ML	35	7.14	92.86
					IN	35	1.43	98.57						IN	35	20.00	80.00
					CAU	36	2.78	97.22						CAU	36	37.50	62.50
							C	T								A	G
**SNPi26**	**I19/-175 C>T**	Intron 19	-	rs4148369	CH	36	91.67	8.33	**SNPi38**	**I28/-45 G>A**	Intron 28	-	rs212087	CH	36	18.06	81.94
					ML	35	91.43	8.57						ML	35	27.14	72.86
					IN	35	81.43	18.57						IN	35	38.57	61.43
					CAU	36	91.67	8.33						CAU	36	58.33	41.67
							C	T								C	T
**SNPe16**	**E20/2651 C>T**	Exon 20	Thr>Met	-	CH	36	100.00	0.00	**SNPi39**	**I29/-83 C>T**	Intron 29	-	-	CH	36	100.00	0.00
					ML	35	98.57	1.43						ML	35	100.00	0.00
					IN	35	100.00	0.00						IN	35	97.14	2.86
					CAU	36	100.00	0.00						CAU	36	98.61	1.39
							C	T								C	G
**SNPi27**	**I20/-59 C>T**	Intron 20	-	-	CH	36	100.00	0.00	**SNPi40**	**I30/-51 G>C**	Intron 30	-	-	CH	36	0.00	100.00
					ML	35	97.14	2.86						ML	35	0.00	100.00
					IN	35	100.00	0.00						IN	35	2.86	97.14
					CAU	36	100.00	0.00						CAU	35	0.00	100.00

* The numbers of SNPs are defined as SNP(SNP locates the region)(In the certain region, the consequent number of the SNP). For example, SNPu1 means the SNP is located in the untranslated region (UTR) and the first SNP in the UTR.

** SNP ID for these SNPs are defined as (Region)/(position number)(major allele)(minor allele). Region shows whether SNP is in an exon (E) or intron (I). Position number of exonic SNP is the mRNA nucleotide position using the translation start site as position 1. For intronic SNP, the position in the genomic sequence is either upstream (-) of using the 5' boundary of the immediate downstream exon as position -1, or downstream (+) using 3' boundary of the immediate upstream exon as position 1. The same principle is employed to the indel and STR. The position is specific to the first nucleotide in the genomic sequence. (reference mRNA ID:NM_004996.2).

*** CH, ML, IN and CAU represent Chinese, Malay, Indian and Caucasian, respectively.

**Table 3:** Frequencies of insertion/deletions (indel) identified at *MRP*1

**No.**	**indel ID**	**Region**	**Amino acid change**	**dbSNP (rs)**	**Population**	**n**	**frequency (%)**	
							**insertion**	**Deletion**
**Indel1**	**5'UTR/-74 14bp indel**	5'UTR	-	-	CH	36	100.00	0.00
					ML	35	100.00	0.00
					IN	35	97.14	2.86
					CAU	36	100.00	0.00
**Indel2**	**I2/17 G indel**	Intron 2	-	-	CH	36	0.00	100.00
					ML	35	0.00	100.00
					IN	35	0.00	100.00
					CAU	36	1.39	98.61
**Indel3**	**I3/-70 T indel**	Intron 3	-	-	CH	36	0.00	100.00
					ML	35	0.00	100.00
					IN	35	0.00	100.00
					CAU	36	1.39	98.61
**Indel4**	**I5/-25 12 bp indel**	Intron 5	-	rs3830390	CH	36	0.00	100.00
					ML	35	0.00	100.00
					IN	35	1.43	98.57
					CAU	36	0.00	100.00
**Indel5**	**I12/-119 G indel**	Intron 12	-	-	CH	36	0.00	100.00
					ML	35	1.43	98.57
					IN	35	0.00	100.00
					CAU	36	0.00	100.00
**Indel6**	**I16/7 6bp indel**	Intron 16	-	-	CH	36	0.00	100.00
					ML	35	1.43	98.57
					IN	35	0.00	100.00
					CAU	36	0.00	100.00
**Indel7**	**I17/-182 5bp indel**	Intron 17	-	-	CH	30	81.70	18.30
					ML	35	74.29	25.71
					IN	29	86.20	13.80
					CAU	30	80.00	20.00
**Indel8**	**I18/-38 AT indel**	Intron 18	-	-	CH	36	0.00	100.00
					ML	35	2.86	97.14
					IN	35	0.00	100.00
					CAU	36	1.39	98.61
**Indel9**	**I24/49 C indel**	Intron 24	-	-	CH	36	0.00	100.00
					ML	35	0.00	100.00
					IN	35	1.43	98.57
					CAU	36	0.00	100.00
**Indel10**	**I29/-13 T indel**	Intron 29	-	rs4148379	CH	36	45.83	54.17
					ML	35	41.43	58.57
					IN	35	61.43	38.57
					CAU	36	75.00	25.00

**Table 4:** Frequencies of GCC trinucleotide repeats at *MRP*1

**Trinucleotide repeats**	**Region**	**Amino acid change**	**dbSNP (rs)**	**GCC repeat No.**	frequency (%)*			
					**CH **(n = 36)	**ML **n = (35)	**IN **n = (35)	**CAU **n = (36)
**5'UTR/-118 GCC repeats**	5'UTR	-	-	7			**18.57**	**12.50**
				8			1.43	
				9	5.56	1.43	4.29	1.39
				10	1.39	4.29		
				11	**25.00**	**20.00**	**21.43**	5.56
				12	9.72	5.71		
				13	**50.00**	**51.43**	**47.14**	**56.94**
				14	4.17	**10.00**	5.71	**19.44**
				15	2.78	7.14	1.43	4.17
				16	1.39			

* Repeat frequencies in bold represent GCC repeats that have relative frequencies (≥ 10%).

**Table 5:** Comparisons of polymorphisms identified in this study with those reported in HapMap/Perlegen

**A**											
			
			HAPMAP				Perlegen				
			
			CHB		CEPH		CHB		EA		
	# common polymorphisms		19		19		14		14		
	
This Study	# non-common polymorphisms	polymorphisms found **in HapMap/Perlegen **but not in this study	25 (22)		23 (22)		1 (0)		1 (0)		
		
		polymorphisms found **in this study **but not in HapMap/Perlegen	41 (26)		41 (26)		46 (28)		46 (26)		

	() refers to number of monomorphic SNPs in that category in that population										

**B**											

**SNP ID**	**dbSNP ID**	**Popu**	**N**	**Our data**		**N**	**HapMap**		**n**	**Perlegen**	

I2/26 G>A	rs8187843			A	G		A	G			
		CH	36	0.00	100.00	45	0.00	100.00			
		CAU	35	7.14	92.86	60	5.83	94.17			
I7/31 G>T	rs8187850			G	T		G	T			
		CH	35	100.00	0.00	45	100.00	0.00			
		CAU	36	98.61	1.39	60	100.00	0.00			
I7/54 C>A	rs903880			A	C		A	C			
		CH	34	1.47	98.53	45	4.44	95.56			
		CAU	36	13.89	86.11	60	25.00	75.00			
I7/64 C>G	rs246232			C	G		C	G		C	G
		CH	34	61.76	38.24	44	55.68	44.32	21	50.00	50.00
		CAU	35	80.00	20.00	60	66.67	33.33	24	52.08	47.92
E8/825 T>C	rs246221			C	T		C	T		C	T
		CH	36	36.11	63.89	45	46.67	53.33	24	56.25	43.75
		CAU	36	13.89	86.11	60	28.33	71.67	22	45.45	54.55
E9/1062 T>C	rs35587			C	T		C	T		C	T
		CH	36	38.89	61.11	45	46.67	53.33	24	58.33	41.67
		CAU	36	31.94	68.06	60	28.33	71.67	24	45.83	54.17
I11/-78 G>A	rs35595			A	G		A	G			
		CH	36	20.83	79.17	45	31.11	68.89			
		CAU	36	13.89	86.11	60	13.33	86.67			
I11/-48 C>T	rs3765129			C	T		C	T		C	T
		CH	36	88.89	11.11	45	88.89	11.11	24	93.75	6.25
		CAU	36	90.28	9.72	58	85.34	14.66	24	85.42	14.58
I12/56 C>T	rs17265551			C	T		C	T		C	T
		CH	35	98.57	1.43	45	95.56	4.44	24	97.92	2.08
		CAU	36	97.22	2.78	60	90.83	9.17	24	89.58	10.42
I12/-85 G>A	rs4148348			A	G		A	G			
		CH	36	8.33	91.67	45	4.44	95.56			
		CAU	36	8.33	91.67	60	7.50	92.50			
I12/-37 A>G	rs35604			A	G		A	G		A	G
		CH	36	83.33	16.67	45	72.22	27.78	24	81.25	18.75
		CAU	36	79.17	20.83	60	82.50	17.50	24	91.67	8.33
E13/1684 C>T	rs35605			C	T		C	T		C	T
		CH	36	83.33	16.67	45	72.22	27.78	24	81.25	18.75
		CAU	36	80.56	19.44	60	82.50	17.50	24	91.67	8.33
E13/1704 C>T	rs8187858			C	T		C	T		C	T
		CH	36	100.00	0.00	45	100.00	0.00	24	100.00	0.00
		CAU	36	98.61	1.39	60	90.83	9.17	24	95.83	4.17
E16/2001 C>T	rs8187863			C		T		T		C	T
		CH	36	100.00	0.00	45	100.00	0.00	24	100.00	0.00
		CAU	36	98.61	1.39	60	98.33	1.67	24	95.83	4.17
E16/2007 C>T	rs2301666			C	T		C	T		C	T
		CH	36	98.61	1.39	45	97.78	2.22	24	97.92	2.08
		CAU	36	100.00	0.00	60	100.00	0.00	24	100.00	0.00
I18/-30 G>C	rs2074087			C	G					C	G
		CH	36	15.28	84.72				24	14.58	85.42
		CAU	36	18.06	81.94				24	4.17	95.83
I21/26 T>C	rs11075296			C	T		C	T		C	T
		CH	36	0.00	100.00	45	0.00	100.00	24	0.00	100.00
		CAU	36	0.00	100.00	60	0.00	100.00	24	0.00	100.00
I22/62 T>C	rs3887893			G	A		G	A			
		CH	36	58.33	41.67	45	50.00	50.00			
		CAU	36	43.06	56.94	59	37.29	62.71			
E28/4002 G>A	rs2239330			A	G		A	G		A	G
		CH	36	8.33	91.67	45	11.11	88.89	24	16.67	83.33
		CAU	36	37.50	62.50	60	29.17	70.83	24	31.25	68.75
I28/-45 G>A	rs212087			A	G		A	G		A	G
		CH	36	18.06	81.94	45	20.00	80.00	24	18.75	81.25
		CAU	36	58.33	41.67	60	39.17	60.83	24	43.75	56.25

**C**											

P-value	Our data			HapMap			Perlegen				
	CH	EA	Both	CH	EA	Both	CH	EA	Both		
Average difference between datasets											

Our data	-	-	-	0.77	0.24	0.26	0.28	0.83	0.71		
HapMap	0.42	2.11	1.27	-	-	-	**0.03**	0.16	**0.02**		
Perlegen	2.54	0.83	0.85	3.48	3.57	3.67	-	-	-		

Note: Cells in the top right triangular section indicate p values of paired-samples t test, whereas those in the lower triangular section contain the average differences in allele frequencies between comparable SNPs between the respective data sets.

**Table 6:** Nucleotide diversity at *MRP*1

**sequence section**	**length(bp)**	**SNPs**	θ*	π*	**π_NS_**/π_**S**_	**Tajima's D**
**Synonymous**	1194	12	16.15	12.62	0.0743	-0.0209
**Nonsynonymous**	822	6	11.73	0.94		-0.0753

*Values of θ and π are listed as value × 10^4^
